# DNA repair pathways as a novel therapeutic strategy in esophageal cancer: A review study

**DOI:** 10.1002/cnr2.1716

**Published:** 2022-09-22

**Authors:** Mohammad Reza Kheyrandish, Seyed Mostafa Mir, Mehdi Sheikh Arabi

**Affiliations:** ^1^ Medical Cellular and Molecular Research Center Golestan University of Medical Sciences Gorgan Iran; ^2^ Metabolic Disorders Research Center Golestan University of Medical Sciences Gorgan Iran; ^3^ Department of Clinical Biochemistry, Faculty of Medicine Golestan University of Medical Sciences Gorgan Iran

**Keywords:** DNA repair pathways, esophageal cancer, therapeutic strategies

## Abstract

Esophageal cancer (EC) is a common malignancy with a poor prognosis worldwide. There are two core pathways that repair double‐strand breaks, homologous recombination (HR) and non‐homologous end joining (NHEJ) and numerous proteins are recognized that affect the occurrence of HR and NHEJ. Altered DNA damage response (DDR) pathways are associated with cancer susceptibility and affect therapeutic response and resistance in cancers. DDR pathway alterations in EC are still poorly understood. Therefore, the identification of alterations in specific genes in DDR pathways may potentially result in novel treatments for resistant cancers, especially EC. In this review, we aimed to focus on different aspects of DNA damage and repair processes in EC. Also, we reviewed new therapeutic strategies via targeting DNA repair machinery components.

## 1 INTRODUCTION

Esophageal cancer (EC) is the eighth most common cancer in the world.^1^, ^2^ It is characterized by a poor prognosis, high mortality, and variation in geographical location.^3^ It is estimated that approximately 17 000 people are diagnosed with EC each year in the United States, of which approximately 16 000 are expected to die.^4^ Esophageal adenocarcinoma (EAC) and esophageal squamous cell carcinoma (ESCC) are the most common types of EC.^5^, ^6^ The 5‐year survival rate for breast and colon cancers is 90%, while it is about 18% for EC, indicating the need to identify more effective treatments.^7^

Esophageal cancer risk factors can be divided into genetic and non‐genetic factors.^8^ Genetic ones include mutations in regenerative genes regulating the cell cycle and differentiation, epidermal growth factor receptor, tyrosine kinase receptor, and factors in the vascular endothelial growth factor signal pathway.^9^ Environmental factors include smoking, age over 50 years, obesity, gastroesophageal reflux disease (GERD), drinking alcohol, and having precancerous cells in esophageal and biliary reflux.^10^

The most current therapeutic strategies for EC include radiotherapy, chemotherapy, and surgery. Besides that, chemo‐radiotherapy or other combinations of such treatments are also used in the treating patients with EC.^11^, ^12^ Among these surgeries are the most commonly accepted method.^13^ Defects in the genome can lead to DNA damage response (DDR) and cell cycle arrest at the checkpoint, followed by DNA repair mechanisms maintaining the stability of the genome.^14^ One type of DNA damage is the double‐strand break (DSB), which can increase the risk of cancer if the repair process is poorly carried out.^15^

Two main DNA repair mechanisms are mostly involved in DSB–namely, non‐homologous end joining (NHEJ) and homologous recombination (HR).^16^ HR repair (HRR), a more perfect and precise one, is carried out through the homologous chromosome so that a healthy strand is used as a pattern for the damaged strand. However, NHEJ only binds to two damaged ends by removing a few nucleotides, which is susceptible to mistakes.^17^ Although the extent of changes in the gene expression of DDR is not thoroughly understood, mutations and loss of function of genes in the DDR system led to susceptibility to cancer. Thus, the gene expression regulation in the DNA repair system can have a great impact on cancer progression and open a new window in cancer treatment.^18^

In this review, we aimed to focus on different aspects of DNA damage and repair processes in EC. Also, we introduced a new therapeutic strategy via targeting DNA repair machinery components. By targeting proteins involved in DNA repair, cancer cells become more sensitive to radiotherapy, which increases the effectiveness of radiotherapy and treatment of cancer patients.^19^

## 2 ESOPHAGEAL CANCER CLASSIFICATION

Two of the most common types of EC are EAC and ESCC. EAC is more common in developed countries, while ESCC highly occurs in developing countries; also, the third type is called small cell carcinoma, which is very rare.^20^, ^21^ Regarding the stages, it should be noted that the first stage is the primary tumor, the second stage is lymph node involvement, and the third stage is metastasis.^22^ EC cancer has no symptoms in the early stages and usually shows symptoms over the age of 50; in other words, the disease has no symptoms until metastasis, so that the death rate is high. Given these reasons, regular check‐ups for those people with high risk and early detection are crucial.^23^

Both types of cancer cells originate from the epithelium lining of the esophagus.^24^, ^25^ There are multiple treatment options for EAC and ESCC, including surgery (i.e., the most common treatment), radiation therapy, chemotherapy, chemoradiation therapy, laser therapy, and electrocoagulation.^26^ Various signs and symptoms of EC, including difficulty in swallowing, weight loss, chest pain, and cough, have been reported. EC usually occurs in old age and is rare in young people.^27^ x‐ray chest, chest computed tomography (CT), esophagoscopy, and positron emission tomography–computed tomography (PET‐CT) is the most widely used methods for the detection of EC.^28^

Esophageal adenocarcinoma originates from the lower third of the esophagus mucus‐secreting glands. Barrett's disease results from the constant exposure of esophageal mucus cells to gastric acid, which is a major risk factor associated with this type of cancer.^29^ Thus, GERD and Barrett's esophagus are the most significant risk factors associated with adenocarcinoma cells. Due to the protective effects of female sex hormones, the incidence of EAC in women is less common than in men.^30^

Esophageal squamous cell carcinoma originates from squamous cells and involves the upper two‐thirds of esophageal tissues.^31^ This type of cancer mostly depends on the lifestyle and results from smoking and alcohol consumption. Approximately half of the cases are due to smoking, and less than half of the cases are due to alcohol consumption. Also, hot drinks, trauma, and nutritional deficiencies can be other causes of this type of cancer.^32^

It should be noted that damage to the DNA repair system is one of the causes of cancer, but the presence of this system in cancer cells causes cancer cells to survive because in radiotherapy and chemotherapy they work through damage to the cell genome That's why sensitizing cancer cells to radiation and chemotherapy drugs can help to eliminate cancer cells.^33^

## 3 DNA REPAIR SYSTEM IN ESOPHAGEAL CANCER

### 3.1 HR Pathway

Homologous recombination is a type of recombination in which fragments are exchanged between identical DNA molecules inherited from parents.^34^ HR is carried out for a variety of purposes, such as recombination during meiosis and repairing of DSBs. When DSBs occur, another homologous strand is used to accurately repair the genome, called the HRR process.^35^

More precisely, the 5'end strand is removed by exonucleases during the resection process. Then, the RAD5 protein causes a 3′ end attack on the same region of the homologous chromosome.^36^ Moreover, the DNA polymerase extends the 3′ end strands of the invading strand, and the other 3 strands are extended by a DNA polymerase on the displaced DNA. Finally, the newly synthesized strand is attached to the 5 exonuclease‐derived strands, a Holliday structure is formed, and the two strands are separated.^37^

The *RAD51* gene is a major component of the HR process and plays a key role in maintaining genome integrity. Interestingly, moderate reductions in the *RAD51* gene have been shown to reduce or inhibit HR levels in EC cells while significantly decrease the expression of this gene is associated with a maximum 50% increase in HR activity. The reason is that moderate silence of this pathway reduces the amount of HR, but complete silence activates other DNA repair pathways; to increase the effect of radiotherapy, it is better to silence this gene moderately.^38^

The *RAD51C* gene is one of the paralogs of the *RAD51* gene.^38^ The *RAD51C* gene is highly expressed in ESCC cells, causing cancer progression, low survival, and increased tumor size via the *p38/Akt* pathway due to the activation of the *RAD51C* pathway through the single‐strand annealing (SSA) mechanism.^4^

#### 3.1.1 Targeting the HR pathway in cancer cells

Regarding the significant roles of HR in cancer cell survival, monotherapy against HR or in combination with other therapies targeting other biological pathways may efficiently reduce cancer cell survival and induce apoptosis in these cells.^39^

The treatment of EC by targeting the regulation of HR and telomerase in EC has interesting results, where telomerase increases HR through the *RAD51* pathway and maintains genome stability. Inhibition of telomerase prevents telomere elongation and also increases *RAD51*. Hybrid inhibition of HR and telomerase induces apoptosis and cell cycle arrest in G2‐M and prevents the growth of cancer cells.^40^

The protein encoded by the *XRCC3* gene is a *RAD51*‐related protein involved in double‐stranded DNA repair and maintains the chromosome structure. The XRCC3 protein has a higher expression rate in ESCC cells than in normal esophageal cells, reducing the effect of chemoradiotherapy; hence, it is possible to increase the efficacy of chemotherapy and radiotherapy by silencing this gene.^41^

Moreover, the *NRAGE* gene, a member of the melanoma‐related antigen family, is significantly expressed in the nucleus of radiotherapy‐resistant esophageal cells. The decreased expression of this gene leads to a fall in the HRR level and so makes cancer cells more sensitive to radiotherapy. This protein forms a ternary structure with RNF8 and BARD1 proteins that are involved in HR regulation.^42^ ATM is another major protein involved in DNA repair, maintaining chromosome stability by increasing HRR. Further, miR‐101 downregulates the expression of ATM and so improves the toxic effects of radiotherapy in ESCC resistant cells.^43^ Another miRNA that can be considered in the diagnosis and treatment of EC is miR‐127‐3p. Also, it can compromise HRR by reducing the expression of the *XRCC3* gene by targeting miRNA 3′ UTR.^44^

In order to increase the effect of proton beam therapy (PBT), chemical agents targeting HR can be used to increase the sensitivity of cancer cells. The combination of Olaparib with PBT can efficiently reduce the resistance of cancer cells to radiation by increasing DSB, thus inducing apoptosis in cancer cells. Poly (ADP‐ribose) polymerase (PARP) is a cellular protein involved in DNA repair converting the single‐stranded DNA break into DSB, which therefore induces HR.^45^ To date, based on our research in this literature review article, there is no clinical trial study about targeting genes that are involved in DNA repair. But two studies about the *PARP* gene, that is one of the genes involved in DNA repair, will be examined in the future. In the first study, Rucaparib would be tested on 220 participants (ClinicalTrials.gov Identifier: NCT04171700). In the second study, a combination of Olaparib, Pembrolizumab, and Paclitaxel will be tested on 71 cancer patients (ClinicalTrials.gov Identifier: NCT04592211).

Bortezomib is an anticancer drug that activates caspase by reducing the expression of *HIF‐1* and *VEGF* genes; this compound delays DNA repair and induces apoptosis; accordingly, it increases sensitivity to radiotherapy.^46^

The *PRA1* gene is a subset of *RPA*, which is involved in HR processes and DNA replication. The increased expression of this gene in EC, colon cancer, etc. causes cell proliferation; also, it induces resistance to radiotherapy. The *RPA1* gene promotes cell proliferation from G1 to the S cell cycle by upregulating *CDK‐4/cyclin‐D* levels.^47^

Also, a bioinformatics study showed that Mirin and NU7441 (i.e., HR and NHEJ inhibitors, respectively) improved the radiotherapy effect and increased DSB. One of the most well‐known instances of HR inhibitors, mirin was developed against nuclease motion of MRE11 and used to impressively inhibit several cancers. Likewise, NU7441, a selective inhibitor for DNA‐PK, blocked the NHEJ pathway and induced DSB and led to increased radio‐sensitivity.^18^

**FIGURE 1:**
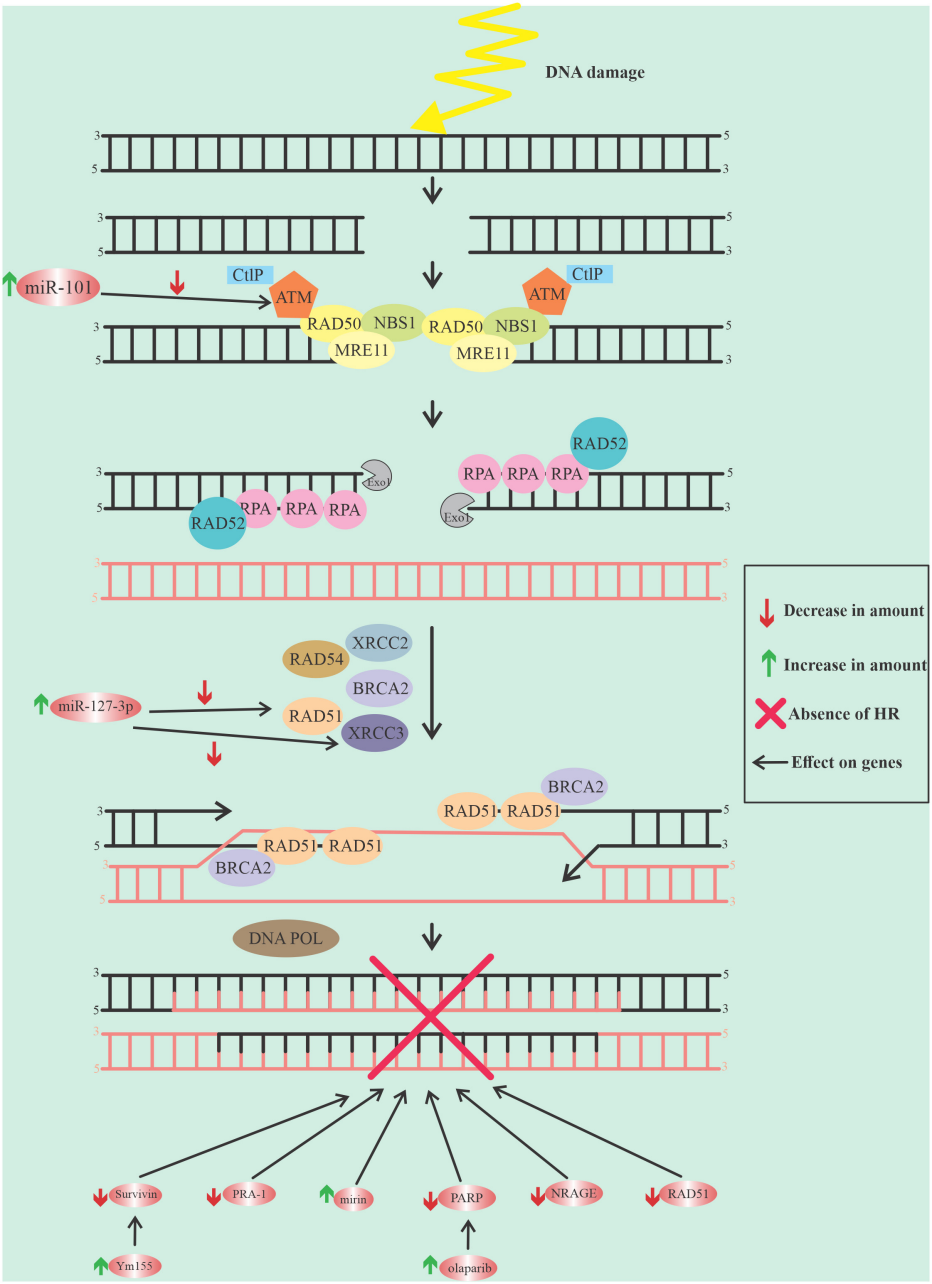
The graphic represents the details of the HR pathway and the factors that involved in interfering this path

Also, studies have shown that mitochondrial superoxide dismutase (MnSOD) has a high potential for genome stability and cancer cell survival. Mice exposed to the plasmid with the *MnSOD* gene had a 7‐fold increase in HR, and when HR occurred, it showed a yellow fluorescent signal.^48^ *YM155* is a small molecule that inhibits survivin. Survivin plays an outstanding role in preventing apoptosis and resistance in all therapy types against cancer cells. *YM155* also increases radiation sensitivity by reducing HRR, see Figure 1.^49^

### 3.2 NHEJ Pathway

Non‐homologous end joining is another type of repair in which the ends of the two strands are connected without the need for another homologous strand.^50^ Non‐homologous end joining initiates the binding of the ku70/80 protein to the damaged end of the chromosome and provides a basis for the binding of subsequent factors, including DNA‐dependent protein kinase catalytic subunit (*DNA‐PKcs), PAXX*, DNA polymerase, and Artemis.^51^ *DNA‐PKcs* has a strong affinity for the *ku70/80* protein and so forms complexes. Then, the *Artemis* nuclease processes the end of the strand by removing a number of nucleotides and facilitates the connection of the ends of the two strands.^52^

Afterward, μ and λ polymerases add a number of nucleotides, and, finally, x‐ray repair cross‐complementing protein 4 (*XRCC4*), *XLF*, and *PAXX* (which together form DNA ligase IV) bind the two broken strands.^53^ The assessment of *XRCC4* gene expression in 92 patients with ESCC showed that in more than 30% of patients, *XRCC4* gene expression decreased. Patients with an increased expression level of this gene have a higher mortality rate than those with decreased expression levels.^54^

#### 3.2.1 Targeting the NHEJ pathway in EC cancer therapy

A therapeutic strategy to increase the efficiency of radiotherapy is the application of histone deacetylases (HDACs), such as valproic acid (VPA). The formation of γH_2_AX by phosphorylation of serine 139 of histones (H_2_AX) is a rapid way to induce DDR through NHEJ. VPA has been shown to decrease DDR levels in EC cancer cells. Also, VPA increases KU70 acetylation and promotes cell apoptosis.^55^

The expression of *HOXC10* has been shown to be higher in EC tumors than in normal cells. The product of this gene associates with cell proliferation by binding to the promoter of the *ERB‐B2* gene and promoting the proliferation of cancer cells. *HOXC10* also accelerates NHEJ by binding to the KU70 protein. Thus, targeting and silencing of this gene can decrease NHEJ and increase radio‐sensitivity.^56^

**FIGURE 2:**
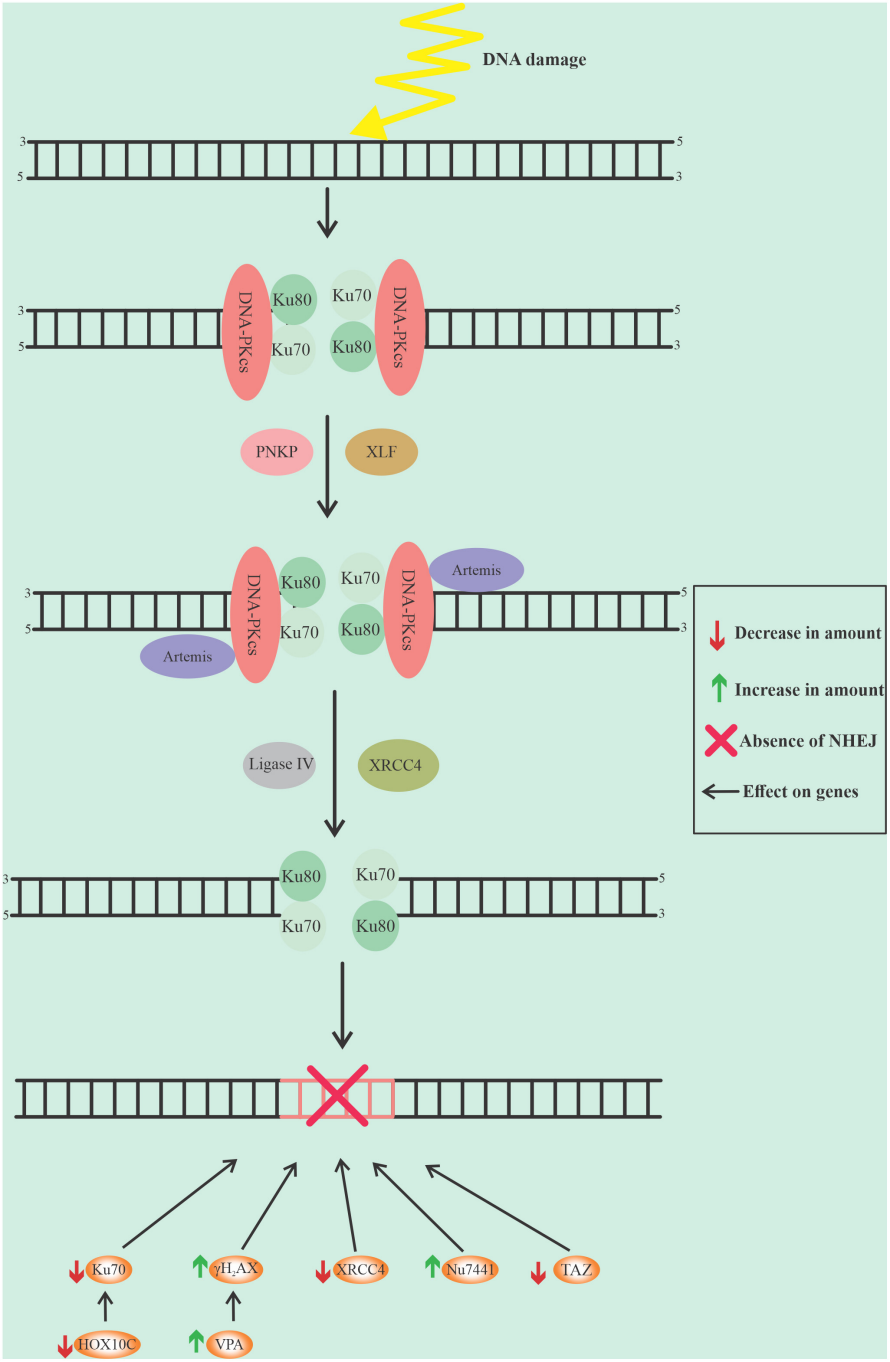
The graphic represents the details of the NHEJ pathway and the factors that involved in reducing this path

A transcription co‐activator with the PDZ binding domain (*TAZ*) has a big impact on the proliferation and survival of cancer cells after radiotherapy. This transcription factor plays a role in DDR by regulating the expression of NHEJ genes, which is controlled by thymine DNA glycosylase (*TDG*). The inhibition of TDG increases the efficiency of radiotherapy and reduces cell growth in EC, see Fig. 2.^57^

## 4 CONCLUSION

Currently, radiotherapy, chemotherapy and chemo‐radiotherapy are treatment options for EC. Also, radiotherapy is one of the most effective ways to treat cancer; in this regard, one of the challenges is the resistance of cancer cells to radiotherapy. Accordingly, we showed that silencing repair genes is necessary and more effective to complete radiotherapy. Indeed, DNA repair machinery can moderate radiotherapy resistance. In this regard, the main targeting components of DNA repair machinery such as RAD51, XRCC3, RPA‐1 in HR and XRCC4, KU70, KU80 in NHEJ can open a new window for treatment of the EC by sensitizing tumor cells to radiotherapy. However, further studies are needed to investigate the development of novel DDR components inhibitors in the EC.

## AUTHOR CONTRIBUTIONS

**Mohammad Reza Kheirandish:** Writing – original draft (equal). **Seyed Mostafa Mir:** Data curation (equal); investigation (equal). **Mehdi Sheikh Arabi:** Supervision (lead); validation (lead); writing – review and editing (lead).

## CONFLICT OF INTEREST

The authors declare that there is no conflict of interest.

## ETHICS STATEMENT

This study was approved by the Ethics Committee of Golestan University of Medical Sciences (IR.GOUMS. REC.1400.072).

## Data Availability

Data sharing is not applicable to this article as no new data were created or analyzed in this study.
